# Attitude, barriers and facilitators to practice-based research: cross-sectional survey of hospital pharmacists in Saudi Arabia

**DOI:** 10.1186/s40545-016-0052-z

**Published:** 2016-02-11

**Authors:** Khizra Sultana, Majed Al Jeraisy, Maha Al Ammari, Rahul Patel, Syed Tabish R. Zaidi

**Affiliations:** King Abdullah International Medical Research Centre, King Abdulaziz Medical City, Ministry of National Guard Health Affairs, P.O. Box: 22490, Riyadh, 11426 Kingdom of Saudi Arabia; Pharmaceutical care services King Abdul Aziz Medical City, Ministry of National Guard Health Affairs, Riyadh, Kingdom of Saudi Arabia; Pharmacy, School of Medicine, University of Tasmania, Hobart, Australia

## Abstract

**Background:**

Little is known about the perceived attitude, barriers and facilitators of Saudi Pharmacists about practice based research. We aimed to measure the attitude, barriers, and facilitators of Saudi hospital pharmacists towards pharmacy practice research.

**Method:**

A cross-sectional survey of hospital pharmacists (*n* = 216) working in King Abdulaziz Medical Cities in Central, Eastern and Western region hospitals was conducted during first week of September, 2013. The survey instrument comprised of six different sections that explored pharmacists previous participation in research, items regarding attitude, perception and willingness to participate, motivators, barriers, different areas of interest for doing research and patient demographics. Quantitative data collected was initially explored using frequency distribution, and descriptive analysis was carried out. Mann-Whitney U and independent samples t-test were used to explore the differences between the study variables.

**Results:**

One hundred and eighty two pharmacists completed the survey yielding a response rate of 84 %. Fifty-nine percent of pharmacists have prior research experience. Pharmacists with research experience were more confident in reading and evaluating research papers (*p* = 0.01), and designing a research study (*p* = 0.001). Pharmacists with previous research experience were also more likely to participate in future research opportunities (*p* = 0.004) and were confident in their research skills (*p* = 0.003). No differences were observed about the perceived value of research, facilitators and barriers to participate in research, between pharmacists with prior research experience and pharmacists who have no prior experience to do research.

**Conclusion:**

Pharmacists in this study were unanimous about the importance of research but showed considerable differences in their confidence to carry out research. There is a need to provide additional support to enable Saudi pharmacists in conducting practice based research.

**Electronic supplementary material:**

The online version of this article (doi:10.1186/s40545-016-0052-z) contains supplementary material, which is available to authorized users.

## Background

Given the amount of medical research knowledge almost doubling every 9 years [1], pharmacists should engage in research to ensure the recency of professional practice [1]. Pharmacy practice research is defined as “a component of health services research that focuses on the assessment and evaluation of pharmacy practice” [2]. Effective pharmaceutical care requires an evidence based practice [3]. Such a practice will not only improve the quality of pharmaceutical care delivered by the pharmacist but will also broaden the scope of pharmacy practice. Traditionally, pharmacy has been slower than medicine and nursing in participating in practice based research (PBR) [4]. This trend has been steadily improving over the last few decades with more pharmacists getting involved in PBR [5]. Research is increasingly advocated and strategically supported by professional pharmacy organizations worldwide. The American College of Clinical Pharmacy (ACCP) encouraged pharmacists to participate in PBR [6]. Further support for such research came from the Agency for Health Care Research and Quality (AHRQ) in the US by the establishment of PBR networks for healthcare professionals [7]. The Canadian Society of hospital pharmacists has also established a Research and Education Foundation to promote the development of research skills amongst the hospital pharmacists [8].

Despite the increasing involvement of pharmacists in PBR over recent years, most published studies have originated from the Western world with relatively fewer reports from Asia and Middle East [9]. Pharmacists practicing in Middle Eastern countries are often perceived to show a lack of interest in research. This is contrary to the findings of a recent Qatari study that found the majority of participants were keen to participate in PBR, though some barriers were identified [10]. Saudi Arabia hosts the greatest number of pharmacists involved in hospital pharmacy. Despite this research output in PBR is limited from Saudi Arabia. As such, it is important to investigate Saudi hospital pharmacist’s views and willingness to participate in PBR. The social cognitive theory states that an individual researcher is influenced by both environmental factors and personal characteristics [11]. The environmental factors include financial reward, support from administration for undertaking research, encouragement from colleagues and presence of a research culture. Personal characteristics include undertaking research for personal satisfaction, personal interest, eagerness to learn about disease management, keenness to provide improved quality of care to patients. Both the environmental factors and personal characteristics play a crucial role in influencing a researcher in carrying out research. Bandura stated ‘what people think, believe and feel affect how they behave’ [11]. If a person has favorable attitude towards research, perceives the importance of research for developing the profession, providing better quality of patient care and is aware of the motivators and barriers to take part in research then this person will be more likely participate in research. Guided by the social cognitive theory, we aim to assess the attitude, motivators and barriers of pharmacists to take part in PBR.

## Methods

### Setting

This study was a part of master thesis completed at Pharmacy, School of Medicine, and University of Tasmania and was carried out at the King Abdul Aziz Medical City (KAMC), in Riyadh, Jeddah and Al Ahsa. KAMC is operated by the Ministry of National Guard Health Affairs (MNGHA) in Saudi Arabia and is one of the largest health organizations in the Kingdom. KAMC in Riyadh, Jeddah, and Al Ahsa have 1025, 531 and 400 patient beds in the Central Region, Western region and Eastern regions of Saudi Arabia, respectively. The Pharmaceutical department is divided into outpatient, inpatient and clinical pharmacy services. In Riyadh, we have 33 clinical pharmacists and 175 pharmacists involved in outpatient, inpatient and discharge counselling services. Jeddah has 11 clinical pharmacists and 75 pharmacists and Al Ahsa has 42 pharmacists and one clinical pharmacist. The Clinical pharmacists at MNGHA perform and undertake all duties related with daily clinical activities which includes medical rounds to determine the necessity of drug therapy, provides input into drug selection and monitoring, developing and evaluation of drug therapy protocols, consults and liaises with physicians with regards to drugs without indications, records recommendations / interventions or other appropriate activity into the medical records, participates in the care provided in the ambulatory clinics like anticoagulation, liver transplant and diabetes, participates in pharmaceutical care services department graduate, undergraduate students and resident training programs etc.

### Survey development

A literature review of relevant published studies identified some studies and survey instruments [10, 12–18]. The survey instruments of Peterson et al. [12], Sanai et al [14] and Rosenbloom et al. [13] seemed most suitable for our present study. Rosenbloom et al instrument comprised of 29 attitudinal statements which assessed the perception of pharmacy practice research, perceived barriers, opportunities and facilitators for pharmacists to take part in research [13]. Sanai’s instrument consisted of two sections where the first section determine previous research involvement of pharmacist and the second section measured the perceptions of pharmacists about participation in research [14]. Peterson et al’s instrument had items on pharmacist’s previous participation into research, perceived value of pharmacy practice research and motivators enabling the participation into research [12]. Items from these instruments were adapted for hospital settings of Saudi Arabia with the addition of some relevant items to provide contextual relevance for Saudi Pharmacists.

A group of 3 experienced pharmacy practitioners and academics evaluated the survey instrument for face and content validity. Based on their feedback, the survey instrument was modified and piloted on five pharmacists prior to its distribution to the study participants. A copy of the survey is available as an Additional file 1.

Briefly, the survey has six distinct sections: The first section sought categorical information about the participants ‘Previous participation in research’ with a ‘yes or no’ to question ‘Have you done research before’. The second section included the definition for Pharmacy Practice Research (PPR) and consisted of 21 items regarding attitude, perception and willingness towards participating in practice based research which was measured on a five point Likert scale. (5=strongly agree,4=agree, 3= neutral, 2=disagree and 1=strongly disagree)

The third section consisted of 10 questions about the factors that motivate the participants to take part in research (measured on a five point Likert scale from ‘strongly agree to strongly disagree’) and the fourth section asked 9 questions related to barriers to take part in research like lack of time, staff, opportunity, interest etc. where respondents we asked to tick all the barriers that applied to them.

The fifth section examined the 7 main areas of interest for doing research like pharmacy administration, hospital pharmacy, therapeutics, pharmacokinetics, pharmacoeconomics, basic science and pharmacy practice. The sixth section explored the participant’s demographics.

### Survey distribution

All pharmacists working at KAMC in Central, Eastern and Western regions were invited through email and staff meetings to take part in this survey. The surveys were delivered during the first week of September 2013 via the internal mail system facilitated by a delegated person at each region. It was a self-administered questionnaire and cover letter was attached to each questionnaire with a description of the aim of the study. A total of 216 surveys were distributed among the three regions; Central-114, Western71 and Eastern 31. A reminder email was sent to all the pharmacists 2 and 4 weeks after the initial distribution. The participants were also reminded to return the completed questionnaire to the delegated personnel. Ethical approval was obtained from the King Abdullah International Medical Research Centre (KAIMRC) ethics committee and University of Tasmania (UTAS) Ethics committee.

### Data analysis

The reliability of the four survey scales was measured using Cronbach’s Alpha. Median and the Inter quartile range was reported for attitude, perception, willingness, motivators and barriers to participation in research. The Mann- Whitney test was used on the no previous research experience (NPRE) pharmacist’s scores to all the items in the instrument to determine, if there were differences in the responses of pharmacists with previous research experience (PRE) compared to those with no previous research experience (NPRE). Independent sample T- test was carried out to test if there was a difference in the mean number of barriers reported between the two groups. An alpha value of 0.05 was used to test statistical significance. The data was analyzed using the SPSS version 22.

## Results

A total of 182 pharmacists returned the survey giving an above average response rate of 84 %. Responses were representative of all three regions including more than 70 % of the pharmacists from each region (Table 1). The analysis was carried out for 166 participants as 16 surveys were incomplete. Three out of four scales to measure attitude, motivators and perceptions of research showed acceptable reliability with Cronbach’s alpha of 0.80, 0.79 and 0.59, respectively whereas the fourth scale to measure willingness to participate in research showed poor reliability with a Cronbach’s alpha of 0.29. Ninety-eight (59 %) of 166 pharmacists who completed the survey reported previous experience with practice based research. The subsequent sections will report the responses from all participants together with the comparison of responses from participants with research experience and no research experience.

**Table 1:** Demographics of the respondents to the survey *n* = 182(%)

Gender	
Male	80 (44)
Female	93 (51.1)
No answer	9 (4.9)
Age	
25	25 (13.7)
25–30	68 (37.4)
31–35	31 (17)
36–40	26 (14.3)
41–45	12 (6.6)
46–50	10 (5.5)
51 and above	4 (2.2)
No answer	6 (3.3)
Qualification	
Diploma	3 (1.6)
Bachelor	98 (53.8)
Master	37 (20.3)
Pharm D	40 (22)
PhD	1 (0.5)
No answer	3 (1.6)
Number of years of pharmacy	
<2	50 (27.5)
2–5	47 (25.8)
6–10	26 (14.3)
>10	57 (31.3)
No answer	2 (1.1)
Job title	
Assistant Director	2 (1.1)
Associate Clinical Pharmacist	9 (4.9)
Clinical Pharmacist	7 (3.8)
Clinical Pharmacy specialist	8 (4.4)
Coordinator	6 (3.3)
Pharmacist 1(<2 year’s experience)	65 (35.7)
Pharmacist II (>2 year’s experience)	76 (41.8)
Supervisor	6 (3.3)
No answer	3 (1.6)
Response rate	
Riyadh (*n* = 114)	103 (90.35)
Jeddah (*n* = 71)	55 (77.46)
Al Hasa (*n* = 31)	24 (77.4)

### Attitude towards research

Table 2 shows the results of attitude towards research scale. Pharmacists in general showed a positive attitude with the majority being supportive of participating in research. Pharmacists were unanimous about the importance of practice based research in determining the best practice and confidence in their ability to understand research, confidence to conduct practice based research was scored high. Significant differences on the scores of various items were observed between pharmacists who indicated previous research experience and those with no research experience (Table 2). Pharmacists with research experience were more likely to enjoy reading research papers (Mann-Whitney U, *z* = −2.040, *p* = 0.041), confident in evaluating research findings (Mann-Whitney U, *z* = −2.583, *p* =0.01) and designing a research study (Mann-Whitney U, *z* = −3.407, *p* = 0.001) when compared to the pharmacists with no research experience.

**Table 2:** Attitude towards research participation

	^a^overall score	^b^PREP	^c^NPREP	*z* value	*P* value
	Median (IQR)^d^	Median (IQR)^d^	Median (IQR)^d^		
I enjoy reading pharmacy practice research studies in the literature.	4 (3, 4)	4 (4, 5)	4 (3, 4)	−2.040	0.041^f^
I would enjoy working on a pharmacy practice research project.	4 (4, 5)	4 (4, 5)	4 (4, 5)	−0.966	0.334
I am confident in my ability to understand research and research terminology related to pharmacy practice.	4 (4, 5)	4 (4, 5)	4 (3–5)	−1.747	0.081
I am confident in my ability to design a pharmacy practice research project.	4 (3, 4)	4 (4, 5)	4 (3, 4)	−3.407	0.001^e^
I am confident in my ability to evaluate research findings in terms of their application to pharmacy practice.	4 (3, 4)	4 (3, 4)	4 (3, 4)	−2.583	0.01^d^
Pharmacy practice research is important in identifying and investigating problems in pharmacy.	5 (4, 5)	5 (4, 5)	4 (4, 5)	−0.490	0.624
Pharmacy practice research is important to pharmacy decision-making.	5 (4, 5)	5 (4, 5)	4 (4, 5)	−0.498	0.619

^a^Attitude was measured on a scale of 1–5 where 1 was ‘strongly disagree’ and 5 was ‘strongly agree’

^b^ *PREP* Previous Research Experience Pharmacists

^c^ *NPREP* Non Previous Research Experience Pharmacists

^f^ *P* value < 0.05

^e^ *P* value < 0.01

^d^ *IQR* Inter quartile range

### Perceived importance of research in pharmacy practice

Table 3 shows the results of response to perceived importance of research in pharmacy practice scale. The perception towards research importance was favorable as majority of the pharmacist perceived research to be important to improve patient care, pharmacy profession and for self-reward. The majority of participants agreed that PBR is relevant to their practice that is important to patient care and should be a high priority for all pharmacists. Similarly, most pharmacists reported that research has influenced their clinical practice. Less than 15 % disagreed with the concept of the importance of research for self-recognition and less than a quarter disagreed with the importance of research for self-satisfaction. There were no significant differences amongst the PRE and NPRE (Table 3).

**Table 3:** Perceived importance of research in pharmacy practice

	^a^overall score	^b^PREP	^c^NPREP	*z* value	*P* value
	Median (IQR)^d^	Median (IQR)^d^	Median (IQR)^d^		
Research should be a high priority for pharmacist.	4 (3, 4)	4 (3, 4)	4 (3, 4)	−0.119	0.905
It is important to be kept informed about research findings.	4 (4, 5)	4 (4, 5)	4 (4, 5)	−0.389	0.697
My daily practice is influenced by evidence based medicine.	4 (3, 4)	4 (3–5)	4 (3, 4)	−0.861	0.389
Research findings are irrelevant to me as a practicing pharmacist.	2 (1–3)	2 (2, 3)	2 (1–3)	−0.217	0.828
Research is important to improve patient care.	5 (4, 5)	5 (4, 5)	5 (4, 5)	−0.692	0.489
Research is important for my recognition.	4 (4, 5)	4 (4, 5)	4 (4, 5)	−0.091	0.927
Research is important for my self-satisfaction.	4 (4, 5)	4 (4, 5)	4 (4, 5)	−0.465	0.642

^a^Attitude was measured on a scale of 1–5 where 1 was ‘strongly disagree’ and 5 was ‘strongly agree’

^b^ *PREP*-Previous Research Experience Pharmacists

^c^ *NPREP* Non Previous Research Experience Pharmacists

^d^ *IQR* Inter quartile range

### Willingness to participate in research

Table 4 shows the results of willingness to participate in research scale. The majority of participants were willing to participate in research and three quarters were against getting paid to conduct research. Most pharmacists surveyed believed that their workload did not permit them to participate in research. Despite this limitation, around 70 % of the participants were interested in doing practice based research with nearly half willing to make time for it. Understandably, pharmacists with previous research experience were more likely to acknowledge the opportunities available for them to participate in research (Mann-Whitney U, *z* = −2.863, *p* = 0.004), indicated they have the necessary skills to do research (Mann-Whitney U, *z* = −2.944, *p* = 0.003) and required less supervision to conduct research (Mann-Whitney U, *z* = −0.001, *p* = 0.001) when compared to pharmacist without research experience.

**Table 4:** Willingness towards research participation

	^a^overall score	^b^PREP	^c^NPREP	*z* value	*P* value
	Median (IQR)^d^	Median (IQR)^d^	Median (IQR)^d^		
There are plenty of opportunities for me to take part in research.	3 (2–4)	3 (2–4)	3 (2, 3)	2.863	0.004^e^
I have the necessary skills to take part in research.	4 (3, 4)	4 (3, 4)	4 (3, 4)	2.944	0.003^e^
I would only participate in research if I am paid.	3 (2, 3)	2 (2, 3)	3 (2, 3)	−0.227	0.821
I would require supervision to do research.	4 (3, 4)	3 (3, 4)	4 (4, 5)	−3.299	0.001^e^
My daily activities prevent me from doing research.	4 (3, 4)	4 (3, 4)	4 (3–5)	−1.487	0.137
I am prepared to make time to do research during working hours.	3 (3–5)	3 (3, 4)	3 (2–4)	−1.857	0.063
I would like to undertake pharmacy based research.	4 (3, 4)	4 (3, 4)	4 (3, 4)	−1.058	0.290

^a^Attitude was measured on a scale of 1–5 where 1 was ‘strongly disagree’ and 5 was ‘strongly agree’

^b^ *PREP* Previous Research Experience Pharmacists

^c^ *NPREP* Non Previous Research Experience Pharmacists

^d^ *IQR* Inter quartile range

^e^ *P* value < 0.01

### Motivators and barriers to take part in research

Table 5 describes the motivators to take part in research. The majority of participants perceived providing the best care for the patient, learning more about disease management and helping the profession to grow as motivators to take part in practice based research. Similarly, most pharmacists reported that they will participate in research because of their own interest and for personal satisfaction. No significant difference was observed between participants with research experience and those with no research experience for any of the items on the motivators scale. Only a little over half of the participants considered financial reward or opportunity to earn Continuing Medical Education hours as motivators to take part in research. More than 70 % of the participants agreed that allocation of specific time to conduct research and support of the administration to do research will motivate them to participate in research.

**Table 5:** Motivators towards research participation

	^a^overall score	^b^PREP	^c^NPREP	*Z* value	*P* value
	Median(IQR)^d^	Median (IQR)^d^	Median (IQR)^d^		
Improve the pharmacy profession.	5 (4, 5)	5 (4, 5)	5 (4, 5)	−0.677	0.498
Provide opportunity to learn more about disease management.	5 (4, 5)	4.5 (4, 5)	5 (4, 5)	−0.185	0.853
Provide enhanced services to patients improve patient care.	5 (4, 5)	5 (4, 5)	5 (4, 5)	−0.242	0.809
Provide financial reward.	4 (3, 4)	5 (4, 5)	4 (3, 4)	−0.242	0.667
Interest in clinical research.	4 (4, 5)	4 (3, 4)	4 (4, 5)	−0.430	0.812
Encouragement from a colleague.	3 (3, 4)	4 (4, 5)	3 (3, 4)	−0.238	0.435
Provide me with Continuing Medical education hours.	4 (3, 4)	4 (3, 4)	4 (3, 4)	−0.780	0.887
Provide me with personal satisfaction.	4 (4, 5)	4 (4, 5)	4 (4, 5)	−0.143	0.560
Availability of replacement for my research time.	4 (3, 4)	4 (3, 4)	4 (3, 4)	−0.582	0.701
To support research.	4 (4, 5)	4 (4, 5)	4 (4, 5)	−0.384	0.81

^a^Attitude was measured on a scale of 1–5 where 1 was ‘strongly disagree’ and 5 was ‘strongly agree’

^b^ *PREP* Previous Research Experience Pharmacists

^c^ *NPREP* Non Previous Research Experience Pharmacists

^d^ *IQR* Inter quartile range

A number of barriers limiting pharmacists’ participation in practice based research were highlighted. The most common barrier reported by most pharmacists was lack of time (63 %) followed by not being aware of opportunity (50 %), lack of support (48 %) and never having been asked to do research (46 %). Pharmacists with PRE reported more barriers than pharmacists with NPRE, though this difference did not reach statistical significance (3.26 vs. 2.95 *p* = 0.461)

## Discussion

Contrary to the anecdotal belief that pharmacists from Middle East are not interested in research, the findings of this study demonstrate that pharmacists are in fact cognizant of the importance of PBR in developing pharmacy practice and are willing to participate in PBR. Pharmacists surveyed appeared to understand the relevance, importance and value of research for their practice, patient care, and profession.

The majority of pharmacists also indicated their willingness to participate in research. Nevertheless, several barriers related to time, awareness of opportunity, lack of support and never being asked to take part in the research were identified.

The percentage of pharmacists with research experience was relatively high (59 %) in the present study compared to the other studies where the proportion of pharmacists with research experience ranges between 9–50 % [4, 10, 14, 18–22]. A possible explanation for such a difference may be the presence of higher number of pharmacists with postgraduate qualifications. For example, more than 40 % of participants in this study hold Masters in pharmacy or PharmD degree. In our study, 69 % of the participants reported their interest in conducting research which is comparable to the recent Qatari study in which 70 % of pharmacists showed interest in being part of PBR [19]. Interestingly, pharmacists in the Middle East seem to show more willingness and interest in conducting research as compared to pharmacists from the United Kingdom for which two separate studies found only 32 to 50 % of the pharmacists were interested to conduct research respectively [20, 22]. A possible explanation for such differences could be because the pharmacists in above two studies were working in the community as opposed to the Middle Eastern studies where pharmacists were mainly working in hospital practice.

In our study more than 50 % of the participants showed confidence in their skills and ability to carry out a research project. Previous studies done in Qatar, UK and Australia showed similar attitudes in the respondent’s confidence to carry out research projects [4, 10, 13, 17]. Seventy eight percent of our participants agreed that they understood research terminology. This is in contrast to previous studies that reported relatively poor knowledge of standard health related research terms among physicians and pharmacists [12, 21]. Consistent with other studies, half of the participants (50.5 %) did not feel that financial incentive would be the reason for them to take part in research [13, 17].

Important motivators for the pharmacist to participate in PBR were the desire to improve the profession, opportunity to learn more about disease management, provide enhanced services to patient care and personal satisfaction. The motivators in our study were similar to studies done with pharmacists and clinicians [12, 18, 23]. More than three quarters of the participants expressed their interest in clinical research which is a recognized predisposing factor to participate in research. A study done with physicians in ambulatory care also reported that clinical interest was a motivator for clinicians to take part as it helps to improve the quality of patient care [23]. The barriers that have been identified in our study to research participation were the lack of time, awareness of opportunity, lack of support and never being asked to take part in research. Lack of time has been reported to be a major barrier in almost all the studies done previously in the literature [12, 15, 17]. However, it was interesting to observe that in our study the pharmacists were willing to make time to do research. Similar to our study Armour et al. and Elkassem et al. found lack of support to be one of the barriers [10, 17]. The findings of Peterson et al. are similar to ours where lack of awareness and never being approached were cited as barriers to take part in research [12]. Additionally, a number of studies have identified confidence, skills, knowledge or training to be a barrier to conduct research [10, 17, 20, 22]. It was interesting to note in our study the participants displayed confidence in their skills, knowledge and ability to carry out research.

### Limitations and future work

To the best of the author’s knowledge, the present research is the first study of Saudi pharmacists’ perceptions about PBR. The present study has an excellent response rate of 84 % which demonstrates the sample was representative of the practice settings. A number of limitations are worth considering. Hospitals studied in the current study were part of MNGHA and differences across the hospitals in Saudi Arabia means that pharmacists working in other hospitals may have different perceptions. Participants were asked to report their general experience of research, a detailed description of research experience would have provided more insights into the kind of experience they have with research and would have been more relevant to the study.

Similarly, a significant number of participants have the postgraduate qualification which is the reason for the positive attitude of most of the pharmacist towards research. One of the limitations of the study was all participants belonged to the MNGHA which represents similar culture across the three regions. Hence, to generalize the responses of the questionnaire to the Saudi pharmacist all over the country was not possible. Future research should look into a survey covering all the major hospitals in the different regions of Saudi Arabia. Additionally, the survey instrument used was not subjected to construct or criterion related validity and one scale in our study show limited reliability.

## Conclusion

In conclusion, this study highlighted that the Saudi pharmacists at MNGHA understood the importance and relevance of research as they expressed strong interest to participate and were willing to invest their time. Nevertheless, interest in research does not correspond to their actual ability to undertake research. Addressing the barriers that were identified in this study can potentially improve the research output of hospital pharmacists in Saudi Arabia.

## Additional file

Additional file 1:
**Survey Instrument.** (DOCX 211 kb)
