# Supplementary material for: Attitude, barriers and facilitators to practice-based research: cross-sectional survey of hospital pharmacists in Saudi Arabia
Source: J Pharm Policy Pract. 2016 Feb 11;9:4. doi: 10.1186/s40545-016-0052-z (PMC4751736; doi:10.1186/s40545-016-0052-z)
Supplement: Additional file 1: — Survey Instrument. (DOCX 211 kb) [file 40545_2016_52_MOESM1_ESM.docx]

Dear Pharmacist,

I am conducting a study to assess the ‘**Attitude, Perception and willingness of Saudi Pharmacist towards Pharmacy Practice Research’**. This is a master’s research project done in collaboration with the University of Tasmania, Australia. As you are a practicing pharmacist, I am inviting you to participate in this research study by completing the attached questionnaire; it will take approximately 5-10 minutes of your time.

This is an anonymous questionnaire and I would like to assure, that all information provided will be utilized for the purpose of research and kept confidential.

I thank you for taking the time to assist me in my educational endeavors. Your participation is voluntary and there is no penalty, if you do not participate.

If you have any questions or concerns about completing the questionnaire or about participating in this study, you may contact me at Ext:….. If you have any questions about your rights as a research subject, you may contact the Institutional Review Board officer (IRB) professor…….

Sincerely,

| **Section 1** |
| --- |

**Q1.Have you done research before?**

Yes

No

| **Section 2** |
| --- |

**PHARMACY PRACTICE RESEARCH** is research that focuses on the practice of pharmacy and establishes a solid evidence base for new pharmacy practices and services.

PLEASE INDICATE YOUR LEVEL OF AGREEMENT WITH EACH OF THE FOLLOWING STATEMENTS RELATING TO PHARMACY PRACTICE RESEARCH BY TICKING THE APPROPRIATE BOX:

| **A) Attitude towards Research** |  |  |  |  |  |
| --- | --- | --- | --- | --- | --- |
|  | **Strongly Agree**  **5** | **Agree**  **4** | **Neutral**  **3** | **Disagree**  **2** | **Strongly**  **Disagree**  **1** |
| 1. I enjoy reading pharmacy practice research studies in the literature |  |  |  |  |  |
| 1. I would enjoy working on a pharmacy practice research project |  |  |  |  |  |
| 1. I am confident in my ability to understand research and research terminology related to pharmacy practice. |  |  |  |  |  |
| 1. I am confident in my ability to design a pharmacy practice research project |  |  |  |  |  |
| 1. I am confident in my ability to evaluate research findings in terms of their application to pharmacy practice**.** |  |  |  |  |  |
| 1. Pharmacy practice research is important in identifying and investigating problems in pharma**c**y |  |  |  |  |  |
| 1. Pharmacy practice research is important to pharmacy decision-making |  |  |  |  |  |
| **B) Perceived value of research** | | | | | |
|  | **Strongly Agree**  **5** | **Agree**  **4** | **Neutral**  **3** | **Disagree**  **2** | **Strongly**  **Disagree**  **1** |
| 1. Research should be a high priority for pharmacist |  |  |  |  |  |
| 1. It is important to be kept informed of the research relevant to the practice of pharmacy |  |  |  |  |  |
| 1. My daily practice is influenced by evidence based pharmacy practice research |  |  |  |  |  |
| 1. Pharmacy research findings are irrelevant to me as a practicing pharmacist |  |  |  |  |  |
| 1. Research is important to improve patient care |  |  |  |  |  |
| 1. Research is important for my recognition |  |  |  |  |  |
| 1. Research is important for my self-satisfaction |  |  |  |  |  |
|  | | | | | |
| **C) Willingness to participate in research** | | | | | |
|  | **Strongly Agree**  **5** | **Agree**  **4** | **Neutral**  **3** | **Disagree**  **2** | **Strongly**  **Disagree**  **1** |
| 1.There are plenty of opportunities for me to take part in research |  |  |  |  |  |
| 2.I have the necessary skills to take part in research |  |  |  |  |  |
| 3.I would only participate in research if I am paid to |  |  |  |  |  |
| 4.I would require supervision to do research |  |  |  |  |  |
| 5.My daily activities prevent me from doing research |  |  |  |  |  |
| 6.I am prepared to make time to do research during working hours |  |  |  |  |  |
| 7.I would like to undertake pharmacy based research |  |  |  |  |  |

| **Section 3** |
| --- |

**Q3.What factors may influence your decision to participate in a research project.**

Please indicate your level of agreement with each of the following statements relating to your participation in a research project by ticking the appropriate box.

| **I will take part in a research project** | **Strongly Agree**  **5** | **Agree**  **4** | **Neutral**  **3** | **Disagree**  **2** | **Strongly**  **Disagree**  **1** |
| --- | --- | --- | --- | --- | --- |
| As, it will help to improve the pharmacy profession. |  |  |  |  |  |
| As, it will provide opportunity to learn more about disease management. |  |  |  |  |  |
| As, it will help to provide enhanced services to patients and to improve patient care. |  |  |  |  |  |
| As, it will provide financial reward. |  |  |  |  |  |
| As, I have Interest in clinical research. |  |  |  |  |  |
| Because of encouragement from a colleague. |  |  |  |  |  |
| As, I it will provide me with CME hours (CME continuing medical education) |  |  |  |  |  |
| As it provides me with personal satisfaction. |  |  |  |  |  |
| If, there is availability of replacement for my research time. |  |  |  |  |  |
| In order to support research activities. |  |  |  |  |  |

| **Section 4** |
| --- |

**Q4.What do you think are your barriers or obstacles in taking part in research? (You may tick all that apply)**

No personal interest

Not enough staff

Not aware of opportunity

Not enough time

Never been asked to

Lack of incentives

Lack of knowledge

Lack of support

Specify___________________

Lack of research

| **Section 5** |
| --- |

**Q5. Area of research that interests you? (Tick all that apply)**

Pharmacy Administration (Quality Management)

Basic Science (Pharmacogenetics, New structural drugs)

Pharmacoeconomics /Epidemiology

Pharmacy practice

Hospital Pharmacy

Therapeutics

Pharmacokinetics

Others________________

**Q6. If you have to choose a clinical study, which area interests you? (Tick all that apply)**

Pediatrics/neonates

ICU

Cardiac

Transplant

Nephrology

Surgery

Internal medicine

Geriatrics

Infectious diseases

Oncology

| **Section 6** |
| --- |

**Demographics**

**Q7. Age**

Less than 25

25-30

31-35

36-40

41-45

46-50

51 and above

**Q8.** **Gender**

Male

Female

**Q9. Qualifications acquired. (Tick all that apply)**

Diploma

Bachelor

Master

Pharm. D

PhD

Other_________

**Q10. Your Job Title:**

Director

Assistant Director

Supervisor

Coordinator

Clinical Pharmacy Specialist

Associate Clinical Pharmacist

Clinical Pharmacist

Pharmacist I

Pharmacist II

**Q11. Have you done any clinical training? (Residency or Fellowship)**

Yes

No

**Q12. Do you have any other board certified qualifications?**

Yes

No

**If yes, specify________________________**

**Q13. Current area of practice**

Ambulatory care

Outpatient setting

Inpatient setting

Oncology

Nephrology

Critical care

Internal Medicine

Pediatrics/Neonatology

Transplant

Surgery

**Q14. Number of years of pharmacy experience in your field:**

<2 2-5

6-10 >10

Q.15. **Please feel free to write any comments about your experience, participation or barriers to participate in research.**

**Thank you for taking time to complete this survey**
